# The CXXC1-IGFBP6 Axis Maintains Corneal Epithelial Differentiation via H3K4me3-Dependent Transcriptional Activation

**DOI:** 10.1167/iovs.67.8.7

**Published:** 2026-07-02

**Authors:** Liqiong Zhu, Chaoqun Chen, Zesong Lin, Jiafeng Liu, Huizhen Guo, Jieying Tan, Ying Huang, Zhancong Ou, Huicong Hu, Jiabi Liang, Rongrong Chen, Jianping Ji, Hong Ouyang, Li Wang

**Affiliations:** 1 State Key Laboratory of Ophthalmology, Zhongshan Ophthalmic Center, Sun Yat-Sen University, Guangdong Provincial Key Laboratory of Ophthalmology and Visual Science, Guangzhou, China; 2 Guangdong Basic Research Center of Excellence for Major Blinding Eye Diseases Prevention and Treatment, Guangzhou, China

**Keywords:** limbal stem/progenitor cells, corneal epithelial cell differentiation, corneal ulcer, CXXC1, IGFBP6

## Abstract

**Purpose:**

To define the role and mechanisms of the epigenetic regulator CXXC1 in corneal epithelial cells (CECs) differentiation.

**Methods:**

Chromatin remodeling–related gene ontology (GO) modules were integrated with RNA sequencing (RNA-seq) to identify CXXC finger protein 1 (CXXC1) as a candidate epigenetic regulator of CECs differentiation. Loss-of-function studies were performed in limbal stem/progenitor cells (LSCs), followed by air–liquid interface–induced differentiation into CECs. Effects of *CXXC1* depletion were assessed by RNA-seq, quantitative real-time PCR, and immunofluorescence. Chromatin immunoprecipitation sequencing (ChIP-seq) for CXXC1 and H3K4me3 was combined with transcriptomics to identify insulin-like growth factor binding protein 6 (IGFBP6) as a direct target. The role and pathological relevance of the CXXC1-IGFBP6 axis were evaluated by *IGFBP6* knockdown and in human corneal ulcer tissues.

**Results:**

CXXC1 was expressed throughout the corneal epithelium and was markedly upregulated during LSC-to-CEC differentiation. *CXXC1* depletion impaired differentiation and reduced expression of CECs markers and key corneal epithelial regulators. ChIP-seq revealed CXXC1 binding at the *IGFBP6* locus, coinciding with H3K4me3 enrichment, whereas *CXXC1* knockdown reduced both H3K4me3 enrichment and IGFBP6 expression. *IGFBP6* knockdown phenocopied CXXC1 loss. Depletion of either gene activated inflammatory, angiogenic, and hypoxia-related gene programs, suggesting abnormal corneal epithelial differentiation and pathological changes. Both proteins were markedly reduced in human corneal ulcer lesions.

**Conclusions:**

CXXC1 maintains CECs differentiation through H3K4me3-dependent activation of IGFBP6 and other epithelial regulators. The CXXC1-IGFBP6 axis is associated with pathological changes in corneal ulcer lesions.

Corneal epithelial cells (CECs) form the outermost layer of the eye (corneal area) and is essential for corneal transparency, barrier function, and visual acuity.^1^^,^^2^ The continuous renewal and regeneration of CECs is supported by limbal stem/progenitor cells (LSCs), which resides in the limbus niche.^3^ LSCs sustain its self-renewal and constantly give rise to transient amplifying cells (TACs), which migrate toward the central cornea and differentiate into mature CECs.^4^ Disruption of LSC-to-CEC differentiation cascade can lead to limbal stem cell deficiency, corneal neovascularization, chronic inflammation, and ultimately severe visual impairment, as observed in chemical injury, ocular surface disorders, and corneal ulceration.^5^

Epigenetic modifications have been shown to directly regulate corneal differentiation, viral keratitis, and corneal wound healing. Sasamoto et al.^6^ revealed that TET2-mediated DNA methylation played a vital role in regulating the expression of corneal differentiation-related genes, including LYPD2, LGALS9C, B4GALT5, and LGALS9. Hypermethylation of KLF4 promoter has been associated with viral keratitis,^7^ and H3K9 methylation has been implicated in transcriptional regulation during corneal epithelial wound healing.^8^ These studies indicate that epigenetic regulation plays a significant role in corneal epithelial function, differentiation, and pathological responses. Nevertheless, the role of histone H3 lysine 4 (H3K4) trimethylation (H3K4me3) and its upstream regulators in the LSC-to-CEC differentiation process has not been systematically explored.

CXXC finger protein 1 (CXXC1, alias: Cfp1), a CpG island-binding protein, is integral component of the SET1/COMPASS histone methyltransferase complex.^9^^,^^10^ By recognizing unmethylated CpG islands and recruiting SET1/COMPASS, CXXC1 directs H3K4 methylation to promoter-proximal regions and thereby shapes the transcriptional landscape.^11^ Recent researches revealed that CXXC1-dependent H3K4me3 modification was required for thymocyte development, macrophage phagocytosis and bactericidal activity, T cell differentiation, and T_H_17-associated autoimmune pathology.^12^^–^^15^ However, whether CXXC1 and CXXC1-mediated H3K4me3 contribute to corneal epithelial differentiation remains unclear. In this study, we identified CXXC1 as an important epigenetic regulator of corneal epithelium. By integrating chromatin remodeling–related Gene Ontology modules with RNA sequencing of human LSCs and differentiated CECs, we pinpoint CXXC1 as a differentiation-associated CpG-binding factor. CXXC1 expression is upregulated during CECs differentiation and that *CXXC1* depletion impairs CECs differentiation, downregulates CECs markers and previously reported corneal epithelial regulators, and activated inflammatory, angiogenesis and hypoxia-related gene programs. Mechanistically, we further identified that CXXC1 maintains appropriate H3K4me3 enrichment at the *IGFBP6* locus and at loci encoding key corneal epithelial regulators (such as *KLF4*, and *IRF1*), thereby promoting their expression. IGFBP6 acts as a direct downstream target of CXXC1 and contributes to CECs differentiation. Furthermore, we found that the aberrant expression of CXXC1 and IGFBP6 was associated with the corneal ulcer. Together, these findings define a CXXC1-IGFBP6 epigenetic axis that supports corneal epithelial identity through H3K4me3-dependent transcriptional activation and links epigenomic dysregulation to pathological corneal epithelial changes and disease.

## Material and Methods

### Samples

Human LSCs were isolated from corneoscleral rims, which were provided by the Eye Bank of Zhongshan Ophthalmic Center (Guangzhou, China, 2023KYPJ126). All experimental were performed as approval by the Ethics Committee of Zhongshan Ophthalmic Center.

### Primary Cell Culture

Primary LSCs were isolated and cultured as follows: the corneoscleral rims were mechanically minced into 2-mm² fragments under a sterile condition, followed by enzymatic digestion with 0.2% collagenase IV (Cat. no. 17104019; Gibco, Waltham, MA, USA) for two hours at 37°C. Subsequently, the tissue fragments were incubated with 0.25% trypsin-EDTA (Cat. no. 25200072; Gibco) for 15 minutes at 37°C. After digestion, the cell solution was neutralized with complete culture medium and spun in a centrifuge at 1000 rpm for five minutes. Then, cells were seeded onto polystyrene tissue culture plates precoated with 2% Matrigel (diluted in DMEM medium; Cat. no. 354230; BD Biosciences, Franklin Lakes, NJ, USA). The culture medium was prepared according to our previous study.^16^

### Corneal Epithelial Cell Differentiation System

LSCs-derived CECs were generated using an air-liquid interface (ALI) differentiation system. The transwell inserts were pre-coated with 30 µg/mL collagen I (Cat. no. C3867; Gibco) for one hour at 37°C. Approximately 2.5 × 10^5^ LSCs per insert were seeded into the transwell. LSCs culture medium was added to both the upper insert and the lower compartment for cell culture, with daily medium replenishment until 100% confluence was achieved. During the CECs differentiation phase, to expose the cell sheet to air, the culture medium in the insert was aspirated, while the medium in the lower compartment was replaced with CECs differentiation medium. After five days, LSCs could be differentiated into mature CECs. The CECs differentiation medium was prepared according to our previous work.^17^ All reagents used for cell culture are listed in Supplementary Table S1.

### Immunofluorescence and Hematoxylin and Eosin (H&E) Staining

Before staining procedure, all samples were prepared as follows: cultured LSCs were fixed with 4% paraformaldehyde (PFA) for 30 minutes at room temperature. Human tissues (normal limbus-cornea tissues and corneal ulcer tissues) or CECs cell sheets were fixed with 10% neutral-buffered formalin for 30 minutes (room temperature) and overnight (4°C), respectively. Then the CECs cell sheets were embedded in the histogel (Cat. no. R904012; Thermo Fisher Scientific, Waltham, MA, USA). After this step, the CECs cell sheets and tissues were dehydrated, embedded in paraffin, and cut into a 5 µm section following a standard procedure.

After de-paraffinization, samples were stained with H&E solution according to a standard protocol. For immunofluorescence staining, deparaffinized samples were first blocked with 0.3% Triton X-100 and 3% BSA solution for one hour at room temperature. After blocking, the samples were incubated with specific primary antibodies overnight at 4°C. The following day, all samples were washed with 1× PBS for five times (10 minutes per time). Then, the samples were incubated with secondary antibodies for two hours at room temperature, followed by washing with 1× PBS for five times (10 minutes per time). Hoechst 33342 (Cat. no. H3570; Thermo Fisher Scientific) were used to stain the cell nuclei. Images were captured with a Zeiss LSM 800 confocal microscope and a Leica DMi8 microscope (Leica, Wetzlar, Germany). Antibodies information were listed in Supplementary Table S2.

### RNA Isolation and Quantitative Polymerase Chain Reaction

The RNeasy kit (Cat. no. DP451; Tiangen, Beijing, China) and the PrimeScript RT Master Mix Kit (Cat. no. RR036B; Takara Biotechnology, Kyoto, Japan) were used for RNA extraction and cDNA synthesis, respectively. For quantitative real-time PCR (qRT-PCR) experiment, cDNA, primers, and iTaq Universal SYBR Green Supermix (Cat. no. 1725124; Bio-Rad Life Science, Hercules, CA, USA) were mixed together, followed by performing the reaction on a QuantStudio 7 Flex system (Life Technologies, Singapore City, Singapore). Target gene expression levels were normalized to GAPDH. Each reaction was done in triplicates. All sequences were listed in Supplementary Table S3.

### Western Blot Analysis

LSCs and CECs were washed twice with cold PBS. Next, cells were lysed in RIPA buffer (Cat. no. P0013B; Beyotime Institute of Biotechnology, Shanghai, China) and protease and phosphatase inhibitor cocktails (Cat. no. 1861281; Thermo Fisher Scientific) to extract total proteins. Protein concentration was determined using the BCA assay (Thermo Fisher Scientific). Each LSCs and CECs group consisted of three biological replicates. All samples were loaded and electrophoresed on SDS-PAGE gels (Cat. no. TSP024-15; Tsingke Biological Technology, Shanghai, China), and then were transferred to a PVDF membrane. The membranes were blocked with 5% BSA in TBST (Tris-buffered saline with 0.1% Tween 20) for two hours. After blocking, the membranes were incubated with CXXC1 (Cat. no. ab198977; Abcam, Cambridge, UK) and GAPDH (Cat. no. GTX100118; GeneTex Inc., Irvine, CA, USA) antibodies overnight at 4°C. After this step, the membranes were washed with TBST for three times, and then incubated with secondary antibody (Cat. no. 7074P2; Cell Signaling Technology, Danvers, MA, USA) for one hour at room temperature. Results were visualized using an ECL reagent and a Bio-Rad detection system, and then were analyzed by ImageJ software.

### Gene Knockdown Experiments

Specific targeting *CXXC1* or *IGFBP6* short hairpin RNAs (*shRNAs*) were designed and cloned into the PLKO.1 plasmid, respectively. Each gene has two individual *shRNA* sequences and was used separately. A *scramble shRNA* with a non-targeting sequence was used as the negative control. After packaging, specific lentivirus and 8 µg/mL polybrene were added to the cultured LSCs. After 36 hours of infection, the culture medium was replaced with medium containing 2 µg/mL puromycin. After 48 hours selection, the positive infected LSCs were further differentiated into CECs using an air-liquid interface (ALI) differentiation system. All *shRNA* sequences are detailed in Supplementary Table S4.

### RNA Sequencing (RNA-Seq)

Total RNA of *scramble-*, *shCXXC1-*, *shIGFBP6*-transfected CECs, and primary LSCs were isolated as described above. RNA libraries were constructed with the VAHTS Universal V6 RNA-seq Library Prep Kit (Illumina, San Diego, CA, USA) and further sequenced on a Novaseq 6000 S4 sequencers (Annoroad Gene Technology Co. Ltd., Beijing, China). The sequencing run was set to paired-end 150-bp reads. RNA-seq reads were mapped to the human reference genome (hg19) using STAR. The TPM values and the differential expression genes were calculated using RSEM and DESeq2, respectively. The differential expression genes list was generated under a condition of adjusted *P* value (*P*adj) < 0.05. Functional analysis was performed using DAVID (Database for Annotation, Visualization and Integrated Discovery, https://david.ncifcrf.gov/home.jsp). For Gene Set Enrichment Analysis (GSEA), the results were defined at false discovery rate (FDR) *q* value ≤ 0.05.

### ChIP-Seq

LSCs, *scramble*-transfected LSCs and *shCXXC1*-transfected LSCs were cultured in 10 cm dishes to a density of 1 × 10^7^ and cross-linked with 1% formaldehyde (Cat. no. 28906; Thermo Fisher Scientific) at room temperature for 10 minutes. The reaction was quenched with 0.125 M glycine (Cat. no. 50046; Sigma-Aldrich, St. Louis, MO, USA) for five minutes. After three washes with cold PBS, the cells were collected and lysed in ice-cold sonication buffer (50 mM HEPES-NaOH, 500 mM NaCl, 1 mM EDTA, 1% Triton X-100, 0.1% SDS, 0.1% Na-deoxycholate) for 10 minutes. The chromatin was sheared to fragments of 200–500 bp using a Covaris M220 focused-ultrasonicator. Sheared chromatin was then incubated with primary antibodies overnight at 4°C, followed by immunoprecipitation with Protein A/G Dynabeads (Cat. no. 10002D/10004D; Invitrogen, Thermo Fisher Scientific). The beads were washed sequentially with sonication buffer, low-salt wash buffer (10 mM Tris-HCl [pH 8.0], 1 mM EDTA, 250 mM LiCl, 0.5% NP-40, 0.5% Na-deoxycholate), and TE buffer (10 mM Tris-HCl, pH 8.0). The immunoprecipitated complexes were eluted and decrosslinked in elution buffer (50 mM Tris-HCl [pH 8.0], 10 mM EDTA, 1% SDS) at 65°C for four hours. The recovered DNA was then treated with proteinase K (Cat. no. AM2546; Invitrogen) and RNase A (Cat. no. EN0531; Invitrogen) at 55°C for one hour and purified using the MinElute PCR Purification Kit (Cat. no. 28006; Qiagen, Hilden, Germany). ChIP-seq libraries were constructed with the VAHTS Universal DNA Library Prep Kit (Cat. no. ND607; Vazyme, Nanjing, China) and sequenced on an Illumina NovaSeq 6000 platform for 150 bp paired-end reads. For each ChIP-seq experiment, two biological replicates were performed using the antibody against H3K4me3 (Cat. no. 9751S; Cell Signaling Technology) or CXXC1 (Cat. no. ab198977; Abcam).

### Statistical Analysis

The statistical analysis between two groups was performed using Student's *t*-test. Results were presented as mean ± SD. A *P*-value less than 0.05 (*P* < 0.05) was considered statistically significant. For the gene set overlap analysis, the enrichment factor and *p*-value were calculated using a one-sided Fisher's exact test based on a background of 57,095 genes.

## Results

### CXXC1 Upregulated During Limbal-to-Corneal Epithelial Differentiation and Marks Corneal Epithelial Identity

**Figure 1.**
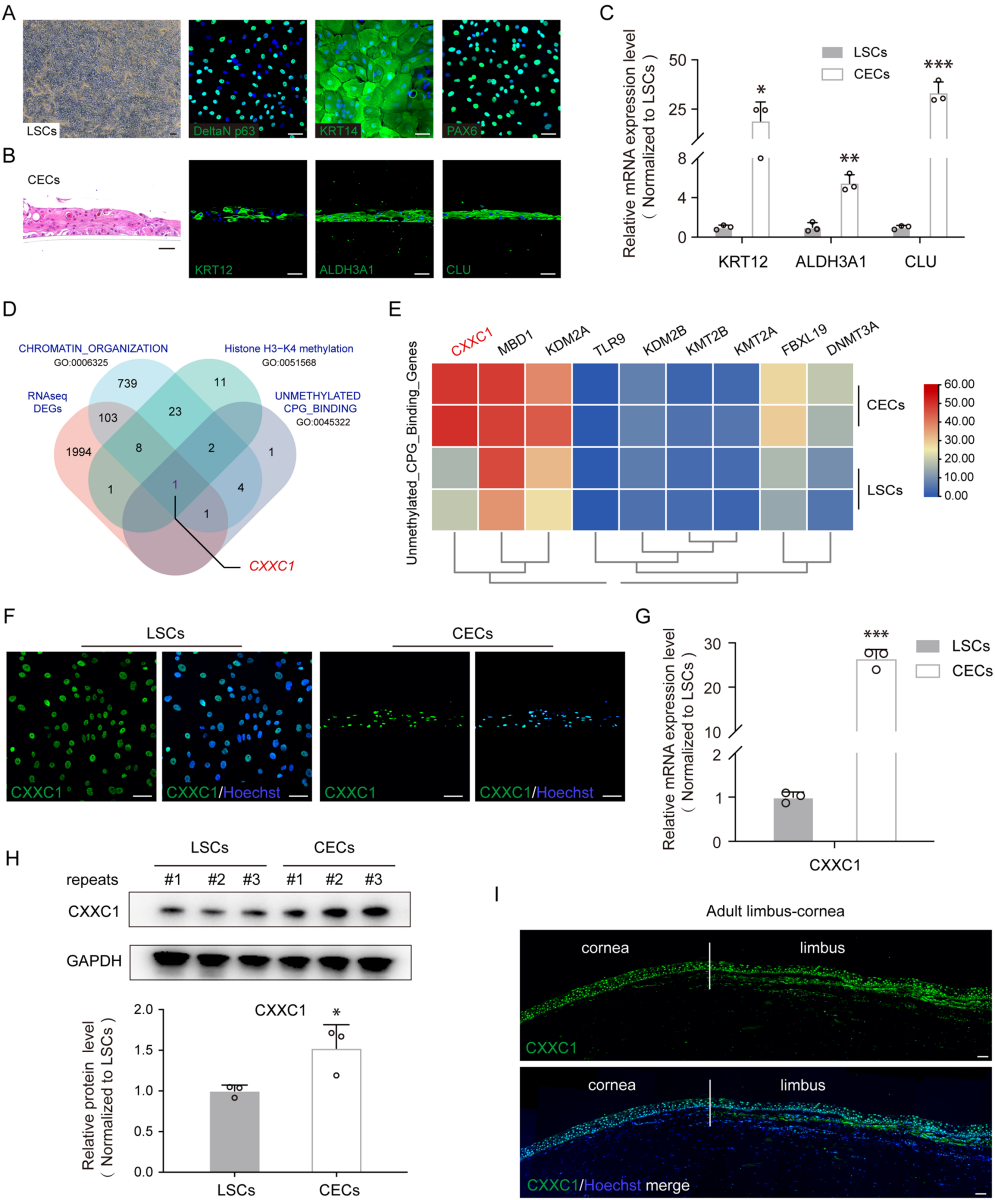
CXXC1 is upregulated during limbal-to-corneal epithelial differentiation and marks corneal epithelial identity. **(A)** Phase-contrast image (*left panel*) and immunofluorescence staining of primary LSCs for the indicated markers. **(B)** H&E staining image (*left panel*) and immunofluorescence staining of air-liquid differentiated CECs for the indicated markers. **(C)** qRT-PCR analysis of the expression of *KRT12*, *ALDH3A1*, and *CLU* in differentiated CECs versus primary LSCs. *n* = 3. **(D)** Venn diagram showing overlapping genes between chromatin remodeling-related GO-defined gene modules and upregulated genes in differentiated CECs. **(E)** Heatmap showing hierarchical clustering of genes annotated under the unmethylated CpG binding GO term (GO:0045322) in LSCs and differentiated CECs based on RNA-seq data. **(F)** Immunofluorescence staining of CXXC1 in LSCs and CECs in vitro. **(G)** qRT-PCR analysis of *CXXC1* expression in LSCs and CECs. *n* = 3. **(H)** Western blot analysis of CXXC1 expression in LSCs and CECs. GAPDH was used as the control. *n* = 3. **(I)** Immunofluorescence staining of CXXC1 in adult human limbus-cornea tissue. *Scale bar*: 50 µm. *P < 0.05, **P < 0.01, ***P < 0.001.

To systematically identify potential epigenetic regulators involved in LSCs-to-corneal epithelial differentiation, human primary LSCs were cultured in vitro and further differentiated into mature CECs using an air-liquid differentiation system as described previously.^17^ The cultured LSCs were characterized using DeltaN p63, KRT14, and PAX6 (Fig. 1A). On air-liquid differentiation, cells acquired a mature corneal epithelial phenotype, as evidenced by markedly increased expression of CECs-specific markers KRT12, ALDH3A1 and CLU (Figs. 1B, 1C). To identify differentiation-associated epigenetic regulators, we performed RNA-seq analysis to define differentially expressed gene set (DEGs) between LSCs and CECs. Given that corneal epithelial fate transition is tightly coupled to chromatin remodeling, particularly promoter-linked H3K4 methylation and CpG island-dependent regulation, we curated three GO-defined gene modules representing this mechanistic axis: Histone H3-K4 methylation (GO:0051568), Chromatin organization (GO:0006325), and Unmethylated CpG binding (GO:0045322). Intersection-analysis among DEGs and the three GO gene sets revealed a limited overlap, within which CXXC1 emerged as the single candidate (Fig. 1D). To further refine candidate selection within the CpG island-dependent regulatory axis identified in Fig. 1D, we focused specifically on genes annotated under the unmethylated CpG binding GO term (GO:0045322). Using RNA-seq data, we performed hierarchical clustering analysis of the expression profiles of all genes within this GO category in LSCs and differentiated CECs. Heatmap visualization revealed that CXXC1 exhibited the most pronounced and consistent upregulation during corneal epithelial differentiation, whereas the majority of other family members displayed relatively modest, unchanged, or heterogeneous expression patterns (Fig. 1E). These data further support CXXC1 as the top candidate epigenetic regulator associated with corneal epithelial lineage commitment.

Consistent with the RNA-seq data, immunofluorescence staining, qRT-PCR and Western blot analyses confirmed that CXXC1 expression was significantly increased in differentiated CECs compared with LSCs in vitro (Figs. 1F–H). Moreover, in adult human limbus–cornea tissue sections, CXXC1 was broadly expressed across all layers of the corneal epithelium, including both limbal and central corneal regions (Fig. 1I). Collectively, these findings identify CXXC1 as a differentiation-associated epigenetic regulator that marks corneal epithelial identity and may play a critical role in corneal epithelial cell maturation.

### CXXC1 Is Required for Transcriptional Programs Driving Corneal Epithelial Differentiation

**Figure 2.**
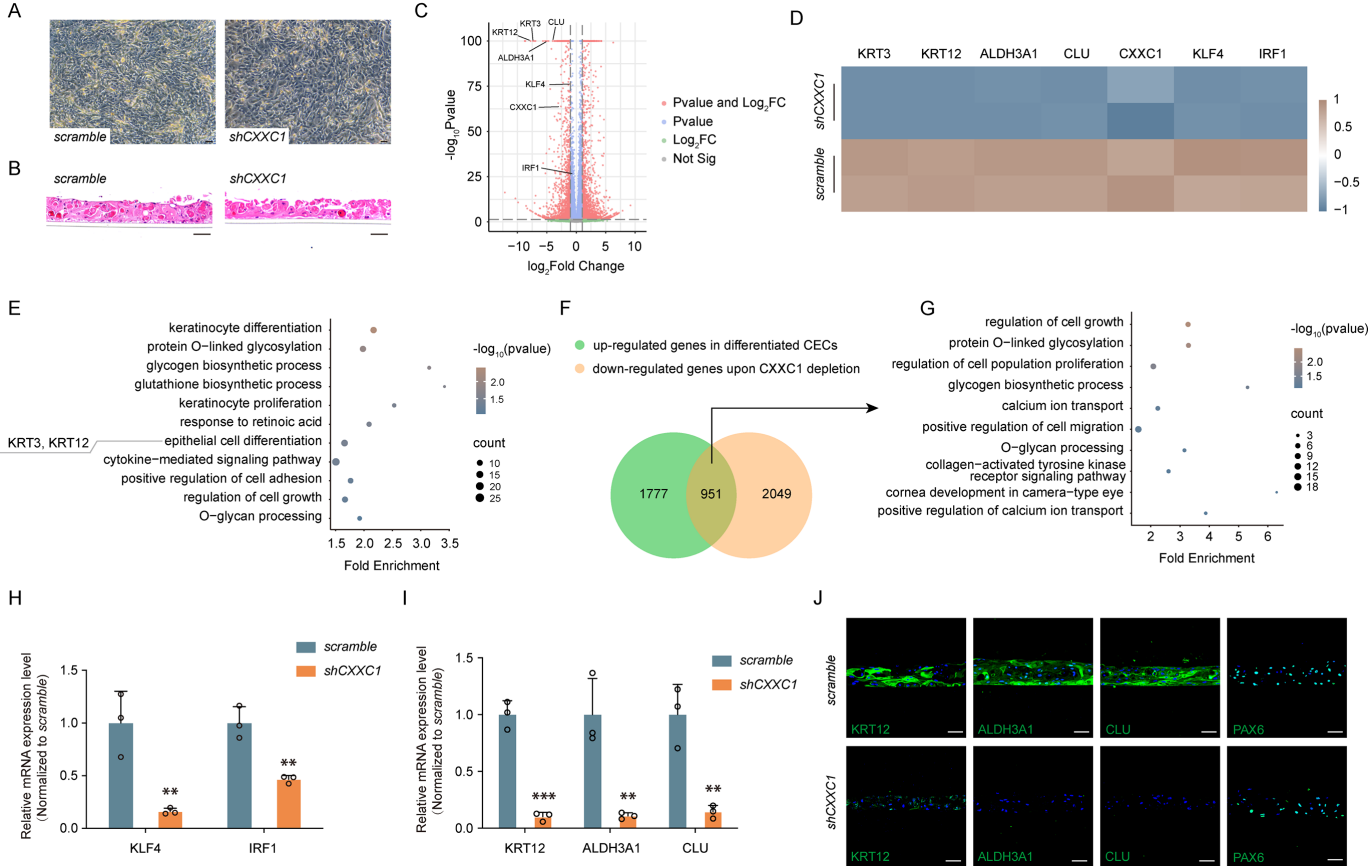
CXXC1 is required for transcriptional programs driving corneal epithelial differentiation. **(A)** Phase-contrast image of *scramble*- and *shCXXC1*-transfected human LSCs. **(B)** H&E staining of CECs derived from *scramble*- and *shCXXC1*-transfected human LSCs using an air-liquid interface differentiation system. **(C)** Volcano plot showing the differentially expressed genes between CECs derived from *scramble*- and *shCXXC1*-transfected LSCs. **(D)** Heatmap showing the *z*-score normalized expression of CXXC1, CEC-specific markers, and previously reported corneal identity- and differentiation-related regulators in CECs derived from *scramble*- and *shCXXC1*-transfected LSCs. The color bar indicates relative expression levels from low to high (z-score, −1 to 1). **(E)** GO BP analysis of genes downregulated in CECs derived from *shCXXC1*-transfected LSCs. **(F)** Venn diagram showing the overlap between genes upregulated during CECs differentiated and genes downregulated in CECs derived from *shCXXC1*-transfected LSCs. Enrichment factor = 6.63; *P* < 1e-300 (one-sided Fisher's exact test). **(G)** GO BP analysis of the overlapping genes shown in **(F)**. **(H, I)** qRT-PCR analysis of CECs-specific markers (*KRT12*, *ALDH3A1*, and *CLU*) and corneal epithelial regulators (*KLF4* and *IRF1*) in CECs derived from *scramble*- and *shCXXC1*-transfected LSCs. *n* = 3. **(J)** Immunofluorescence staining of CECs-specific markers and PAX6 in CECs derived from *scramble*- and *shCXXC1*-transfected LSCs. *Scale bar*: 50 µm. **P < 0.01, ***P < 0.001.

To investigate the functional role of CXXC1 during corneal epithelial differentiation, *CXXC1* was depleted in primary human LSCs using *shRNAs*,followed by induction of differentiation into CECs using an air-liquid interface system. Under the undifferentiated condition, phase-contrast microscopy revealed that *shCXXC1*-transfected LSCs tended to exhibit a larger cell size compared with *scramble*-transfected LSCs (Fig. 2A). In contrast, differentiated *shCXXC1* CECs displayed morphological changes relative to the *scramble* group by H&E staining, characterized by looser cell-cell contacts, a more irregular cellular appearance, and decreased cellular density (Fig. 2B; Supplementary Fig. S1A). RNA-seq analysis demonstrated that depletion of *CXXC1* in CECs led to downregulation of multiple genes associated with corneal epithelial differentiation and epithelial regulation, including KRT3, KRT12, ALDH3A1, CLU, KLF4 and IRF1 (Figs. 2C, 2D). GO BP analysis indicated of the downregulated genes showed significant enrichment for epithelial cell differentiation, keratinocyte differentiation, and positive regulation of cell adhesion (Fig. 2E), consistent with impaired acquisition of corneal epithelial identity upon CXXC1 loss.

To further assess the contribution of CXXC1 to differentiation-associated transcriptional programs, we performed an integrative analysis comparing genes upregulated during CECs differentiation with genes downregulated following *CXXC1* depletion. This analysis identified 951 co-regulated genes (Fig. 2F). GO BP analysis of these overlapping genes revealed enrichment in cornea development in camera-type eye, glycogen biosynthetic process, calcium ion transport, positive regulation of cell migration and regulation of cell population proliferation/cell growth (Fig. 2G), further supporting the role for CXXC1 in coordinating transcriptional programs essential for corneal epithelial differentiation and function.

To validate these transcriptomic findings, we examined the expression of representative CECs differentiation-associated genes and epithelial regulators. The qRT-PCR and immunofluorescence staining results showed that depletion of *CXXC1* led to significantly reduced the expression of KLF4, IRF1, KRT12, ALDH3A1 and CLU (Figs. 2H–J). Additionally, three unchanged genes (*EHF*, *ELF3*, and *FOXC1*) and three upregulated genes (*KRT8*, *TGFB1*, and *ITGB4*) were also validated by qRT-PCR, confirming the reliability of the RNA-seq data (Supplementary Figs. S1B, S1C). Notably, qRT-PCR analysis of LSCs marker genes (*KRT14*, *PAX6*, and *p63*) showed no significant changes upon *CXXC1* knockdown in CECs (Supplementary Fig. S1D), indicating that *CXXC1* depletion specifically affects differentiation-related genes without compromising LSCs identity. Collectively, these results suggested that CXXC1 is required for activating transcriptional programs that drive corneal epithelial differentiation.

### CXXC1 Sustains Corneal Epithelial Identity via H3K4me3-Dependent Transcriptional Activation

**Figure 3.**
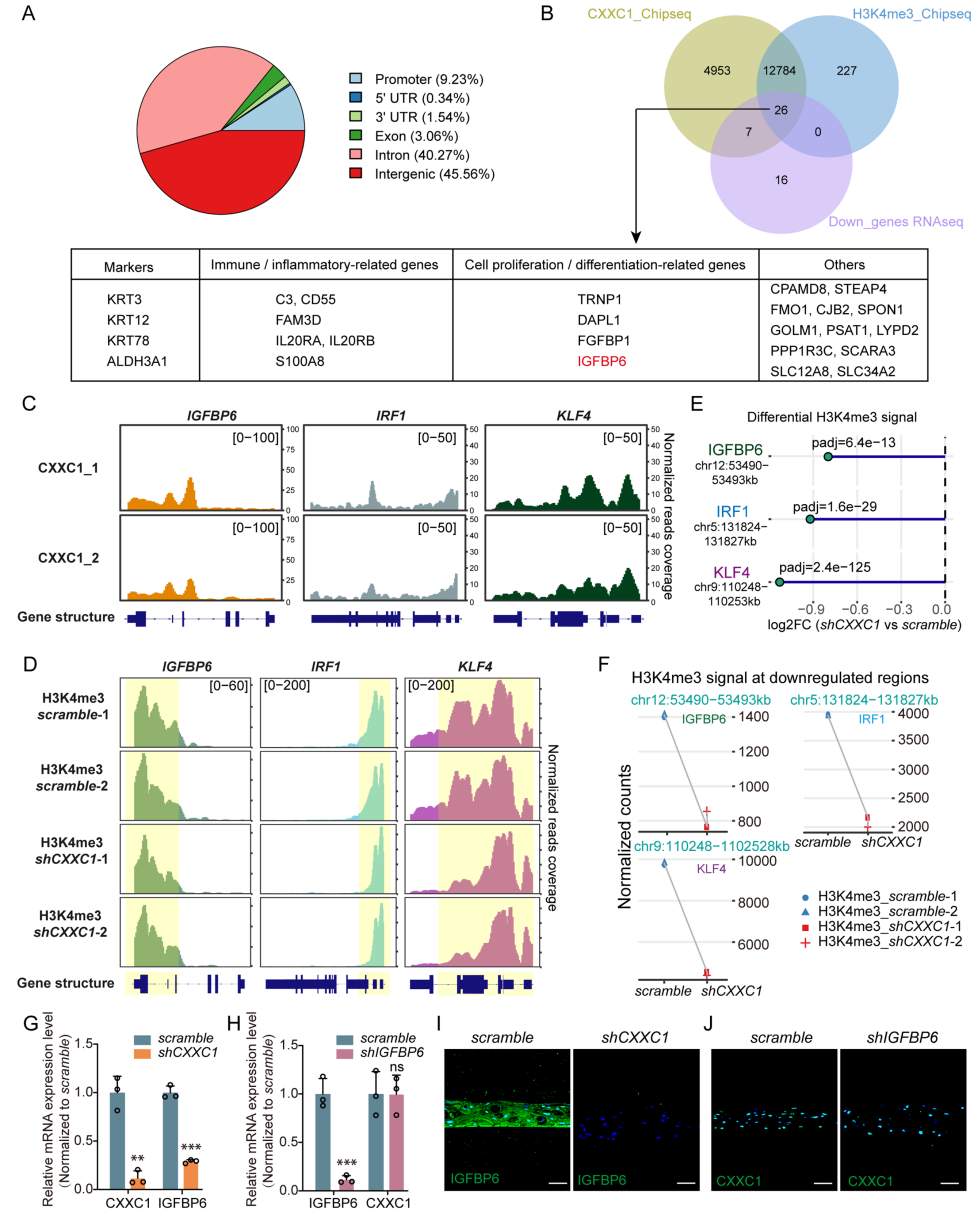
CXXC1 maintains corneal epithelial identity by sustaining H3K4me3 at IGFBP6 and other epithelial regulators. **(A)** Genome-wide distribution of CXXC1-binding sites in primary human LSCs by ChIP-seq. **(B)** Venn diagram showing overlapping genes among CXXC1 binding peaks in LSCs, H3K4me3 signals in LSCs, and downregulated genes in CECs derived from *shCXXC1*-transfected LSCs. **(C, D)** Genome browser tracks showing CXXC1-binding peaks and H3K4me3 enrichment at the indicated gene loci in LSCs. **(E)** Lollipop plot summarizing candidate regions associated with genes downregulated on *CXXC1* knockdown in *scramble* and *shCXXC1* LSCs. **(F)** Region-level plots showing normalized H3K4me3 signals across representative loci in *scramble* and *shCXXC1* LSCs. **(G, H)** **The** qRT-PCR analysis of the expression levels of *CXXC1* and *IGFBP6* in *scramble*-, *shCXXC1*- and *shIGFBP6*-transfected CECs. *n* = 3. **(I, J)** Immunofluorescence staining of IGFBP6 and CXXC1 in CECs derived from *scramble*-, *shCXXC1*- and *shIGFBP6*-transfected LSCs. *Scale bar*: 50 µm. **P < 0.01, ***P < 0.001.

Given that CXXC1 is a key epigenetic regulator, we next sought to elucidate its mechanism of action in CECs differentiation process. Genome-wide CXXC1 ChIP-seq analysis in primary human LSCs revealed that CXXC1-binding sites were broadly distributed across the genome. The majority of CXXC1 peaks localized to intronic (40.27%) and intergenic regions (45.56%), whereas a smaller proportion mapped to promoters (9.23%), exons (3.06%), and untranslated regions (5′UTR, 0.34%; 3′UTR, 1.54%) (Fig. 3A). To identify high-confidence H3K4me3 modification sites, we performed ChIP-seq in two independent biological replicates and utilized the intersection of their peak sets (MACS2, *q* < 0.001) for subsequent overlap analysis with CXXC1 binding sites (Fig. 3B). This integrative analysis identified 26 overlapping genes that were directly bound by CXXC1, exhibited reduced H3K4me3 upon *CXXC1* depletion, and were transcriptionally downregulated in differentiated CECs (Fig. 3B).

GO BP analysis classified these overlapping genes into distinct functional categories, including immune/inflammatory responses and cell proliferation or differentiation. Among the four genes associated with cell proliferation or differentiation, IGFBP6 is notable as a purely secreted protein, suggesting its potential role as a functional mediator linking epigenetic regulation to extracellular signaling in corneal epithelium. Based on these features, IGFBP6 was selected for further mechanistic investigation (Fig. 3B).

Genome browser visualization demonstrated prominent CXXC1 enrichment at transcription start site-proximal regions of the IGFBP6 as well as other key corneal epithelial regulators, including IRF1 and KLF4 loci, in primary LSCs (Fig. 3C). CXXC1-binding regions closely coincided with strong H3K4me3 signals in *scramble* control cells, whereas *CXXC1* depletion resulted in markedly reduction of H3K4me3 enrichment at these loci (Figs. 3D–F), indicating that CXXC1 is required to sustain active H3K4me3 deposition at a subset of corneal epithelial regulators.

Consistent with these epigenomic changes, qRT-PCR and immunofluorescence staining confirmed that depletion of *CXXC1* reduced the expression of *IGFBP6* in differentiated CECs (Figs. 3G, 3I). In contrast, no significant change in *CXXC1* expression was detected after knocking down *IGFBP6* in CECs (Figs. 3H, 3J), placing CXXC1 upstream of IGFBP6 in this regulatory cascade. Together, these findings indicated that CXXC1 act as an upstream epigenetic gatekeeper that directly promotes IGFBP6 transcription through H3K4me3-dependent activation in LSCs, thereby contributing to the maintenance of corneal epithelial identity.

### IGFBP6 Is a Direct Downstream Effector of CXXC1 That Promotes CEC Differentiation

**Figure 4.**
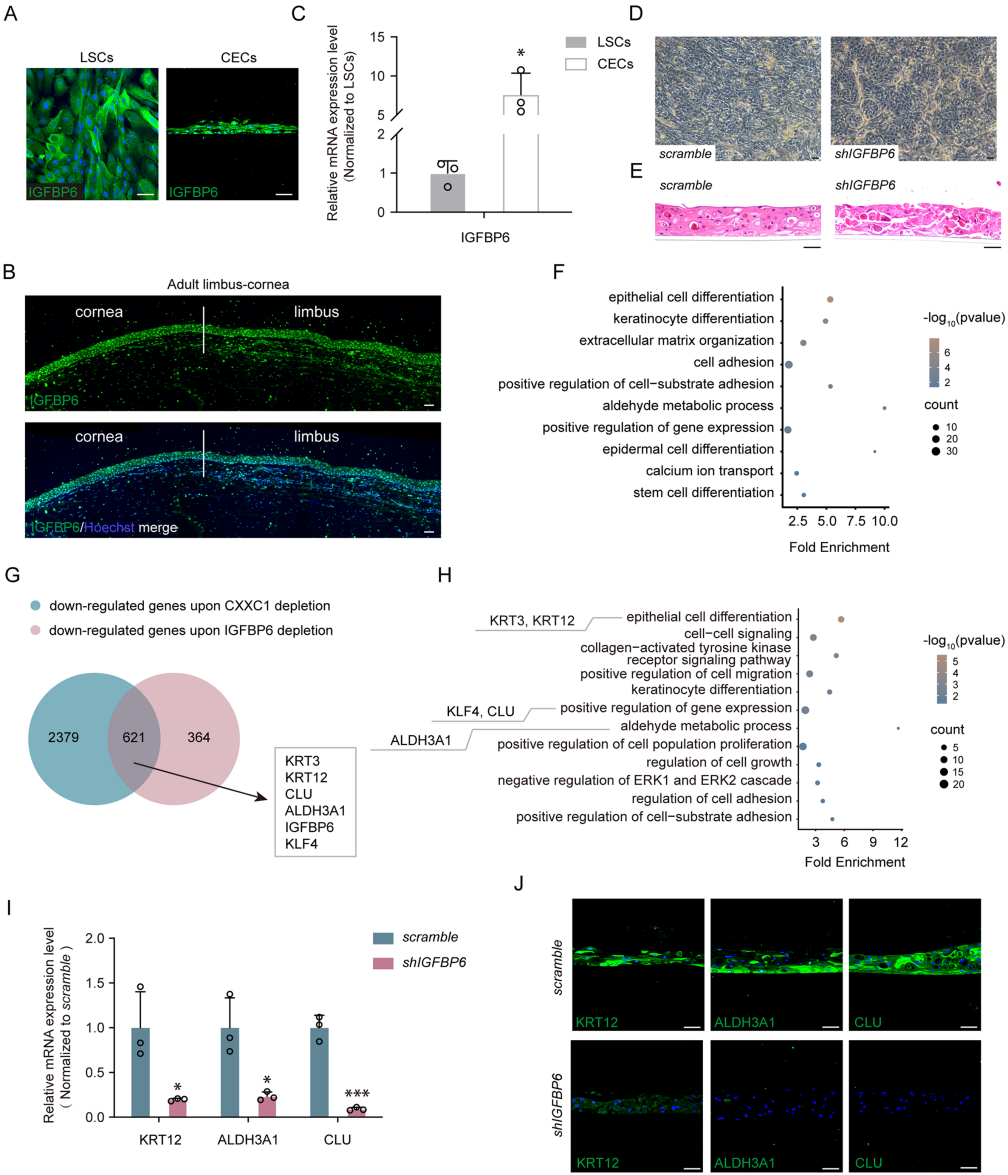
IGFBP6 functions as a downstream effector of CXXC1 to promote corneal epithelial differentiation. **(A, B)** Immunofluorescence staining of IGFBP6 in LSCs, CECs and adult human limbus-cornea tissue. **(C)** QRT-PCR analysis of *IGFBP6* expression in LSCs and CECs. *n* = 3. **(D)** Phase-contrast image of *scramble*- and *shIGFBP6*-transfected human LSCs. **(E)** H&E staining of CECs derived from *scramble*- and *shIGFBP6*-transfected LSCs. **(F)** GO BP analysis of genes downregulated in CECs derived from *shIGFBP6*-transfected LSCs. **(G)** Venn diagram showing overlapping genes downregulated in CECs derived from *shCXXC1*- and *shIGFBP6*-transfected LSCs. **(H)** GO BP analysis of the overlapping genes in (G). **(I)** The qRT-PCR analysis of the CECs-specific marker expression in CECs derived from *scramble-* and *shIGFBP6*-transfected LSCs. *n* = 3. **(J)** Immunofluorescence staining of CECs-specific markers in CECs derived from *scramble-* and *shIGFBP6*-transfected LSCs. *Scale bar*: 50 µm. *P < 0.05, ***P < 0.001.

To further investigate the role of IGFBP6 in corneal epithelium, we first examined its expression in vitro and in vivo. Immunofluorescence staining revealed that IGFBP6 expressed in both the cultured LSCs and differentiated CECs (Fig. 4A). In adult human limbus-cornea tissue, IGFBP6-positive cells were detected throughout the corneal epithelium, including both and limbus and central corneal regions, consistent with the in vitro findings (Fig. 4B). Notably, the *IGFBP6* mRNA expression level increased 7.6-fold in differentiated CECs compared with LSCs (Fig. 4C). These findings suggested that IGFBP6 might participate in regulating corneal epithelial differentiation.

To further explore the function of IGFBP6, we knocked down its expression in LSCs and subsequently induced these cells to differentiate into CECs using air-liquid interface system. Under the undifferentiated condition, phase-contrast microscopy revealed that *shIGFBP6*-transfected LSCs tended to exhibit a larger cell size and showed a tendency to form cell clusters compared with *scramble*-transfected LSCs (Fig. 4D). In contrast, after differentiation, *shIGFBP6*-transfected cells displayed evident morphological changes compared with the *scramble* group, including looser cell-cell contacts, more irregular cell morphology, and reduced cellular density, as revealed by H&E staining (Fig. 4E; Supplementary Fig. S1A). These changes were highly similar to those observed in *shCXXC1* cells. Transcriptomic results revealed that the downregulated genes in CECs derived from *IGFBP6*-transfected LSCs were mainly enriched in biological processes related to epithelial cell differentiation, keratinocyte differentiation, extracellular matrix organization, cell adhesion, calcium ion transport, and positive regulation of gene expression (Fig. 4F).

Given that IGFBP6 was identified as a direct transcriptional target of CXXC1, we next compared the transcriptional consequences of *IGFBP6* and *CXXC1* depletion. Integrated analysis revealed 621 genes that were commonly downregulated in CECs derived from *shCXXC1*- and *shIGFBP6*-transfected LSCs, including key CECs differentiation-associated genes and epithelial regulators such as KRT3, KRT12, ALDH3A1, CLU and KLF4 (Fig. 4G). GO BP analysis revealed that these overlapping genes were mainly enriched in epithelial cell differentiation, keratinocyte differentiation, positive regulation of gene expression, aldehyde metabolic process, regulation of cell growth/cell adhesion/cell migration, as well as negative regulation of ERK1 and ERK2 cascade (Fig. 4H).

Consistent with these transcriptomic changes, qRT-PCR and immunofluorescence staining confirmed that depletion of *IGFBP6* markedly reduced the expression of mature CECs-specific markers, including KRT12, ALDH3A1 and CLU in differentiated CECs (Figs. 4I, 4J). Taken together, these results demonstrate that IGFBP6, a direct downstream target of CXXC1, contributed to CECs differentiation and maintenance of epithelial identity.

### Disruption of the CXXC1-IGFBP6 Axis Induces Pathological Corneal Epithelial Changes and Is Associated With Corneal Ulcer Lesions

**Figure 5.**
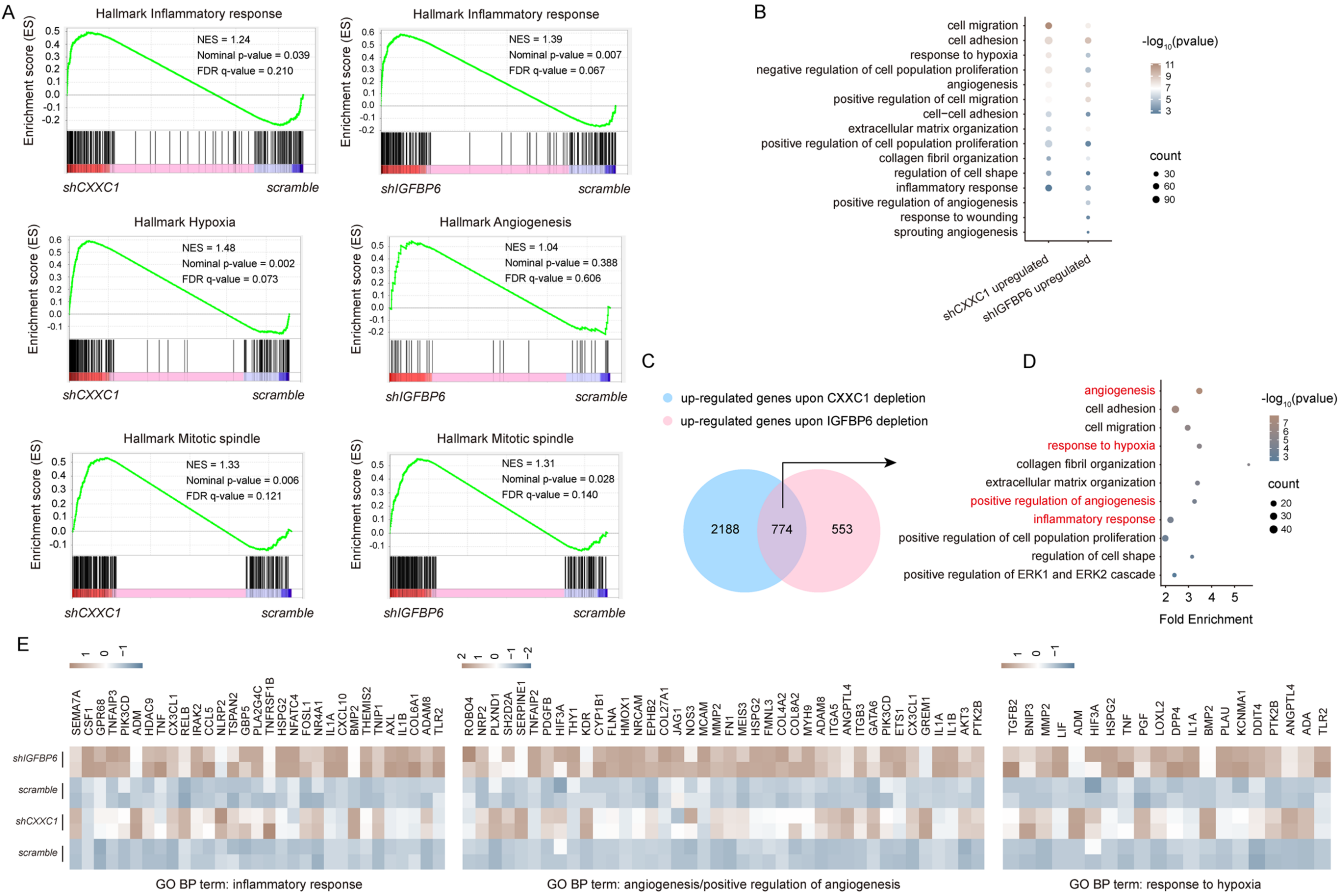
Depletion of *CXXC1* and *IGFBP6* induces pathological corneal epithelial changes. **(A)** GSEA analysis showing the enriched hallmark gene sets in CECs following depletion of *CXXC1* or *IGFBP6* compared with *scramble* controls. **(B)** GO BP analysis of the upregulated genes induced by depletion of *CXXC1* or *IGFBP6* in CECs. **(C)** Venn diagram showing the overlap of upregulated genes in *CXXC1*-depleted CECs and *IGFBP6*-depleted CECs. **(D)** GO BP analysis of the overlapping genes shown in (C). **(E)** Heatmap showing the representative enriched genes in selected biological terms in *scramble*-, *shCXXC1*-, and *shIGFBP6*-derived CECs.

To determine the role of the CXXC1-IGFBP6 axis in corneal epithelial differentiation and its pathological relevance, we analyzed the transcriptional changes induced by depletion of *CXXC1* or *IGFBP6* in differentiated CECs. Gene set enrichment analysis (GSEA) analysis revealed that depletion of either *CXXC1* or *IGFBP6* led to significant activation of hallmark gene sets related to inflammatory response, hypoxia, angiogenesis, and mitotic spindle (Fig. 5A). Consistently, GO BP analysis showed that these upregulated genes showed markedly enrichment in inflammatory response, angiogenesis, response to hypoxia, response to wounding, and positive/negative regulation of cell proliferation (Fig. 5B).

Comparative analysis further identified 774 overlapping upregulated genes upon *CXXC1* and *IGFBP6* depletion (Fig. 5C). Similarly, these overlapping genes were mainly enriched in inflammatory response, angiogenesis/positive regulation of angiogenesis, response to hypoxia, and positive regulation of cell population proliferation/ERK1 and ERK2 cascade (Fig. 5D). Heatmap visualization further illustrated coordinated activation of representative genes involved in inflammatory response, angiogenesis, and hypoxia signaling in CECs lacking *CXXC1* or *IGFBP6* (Fig. 5E). Collectively, these results indicate that loss of the CXXC1-IGFBP6 axis induces a corneal epithelial instability program characterized by inflammatory, angiogenesis, and hypoxia.

**Figure 6.**
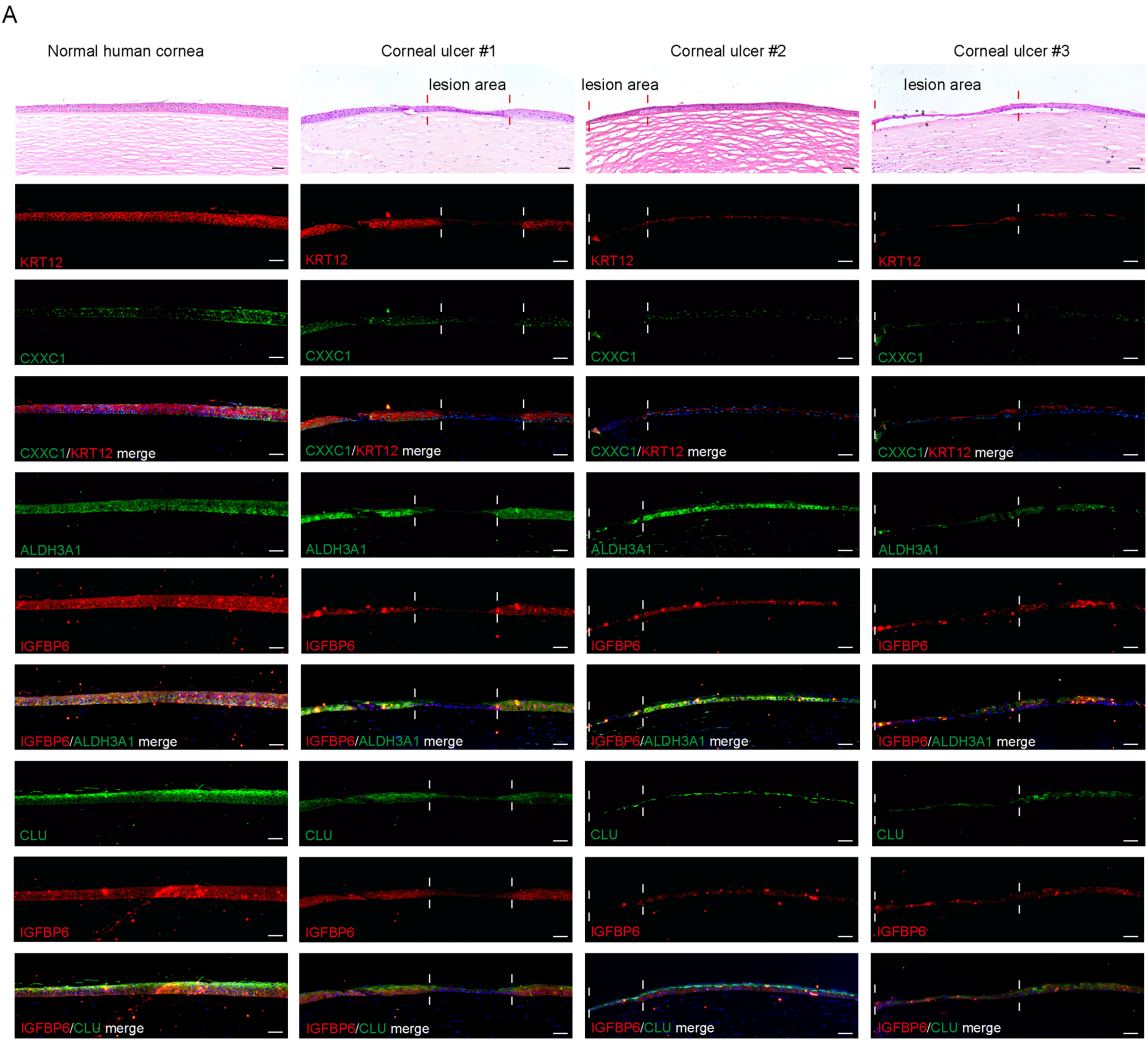
Loss of CXXC1 and IGFBP6 is associated with corneal ulcer lesions. (A) H&E staining and immunofluorescence staining for CXXC1, IGFBP6, KRT12, ALDH3A1, and CLU in adult human corneal ulcer sections and normal human cornea control. *n* = 3 corneal ulcer samples; one normal human cornea sample was included as a control. *Scale bar*: 50 µm.

**Figure 7.**
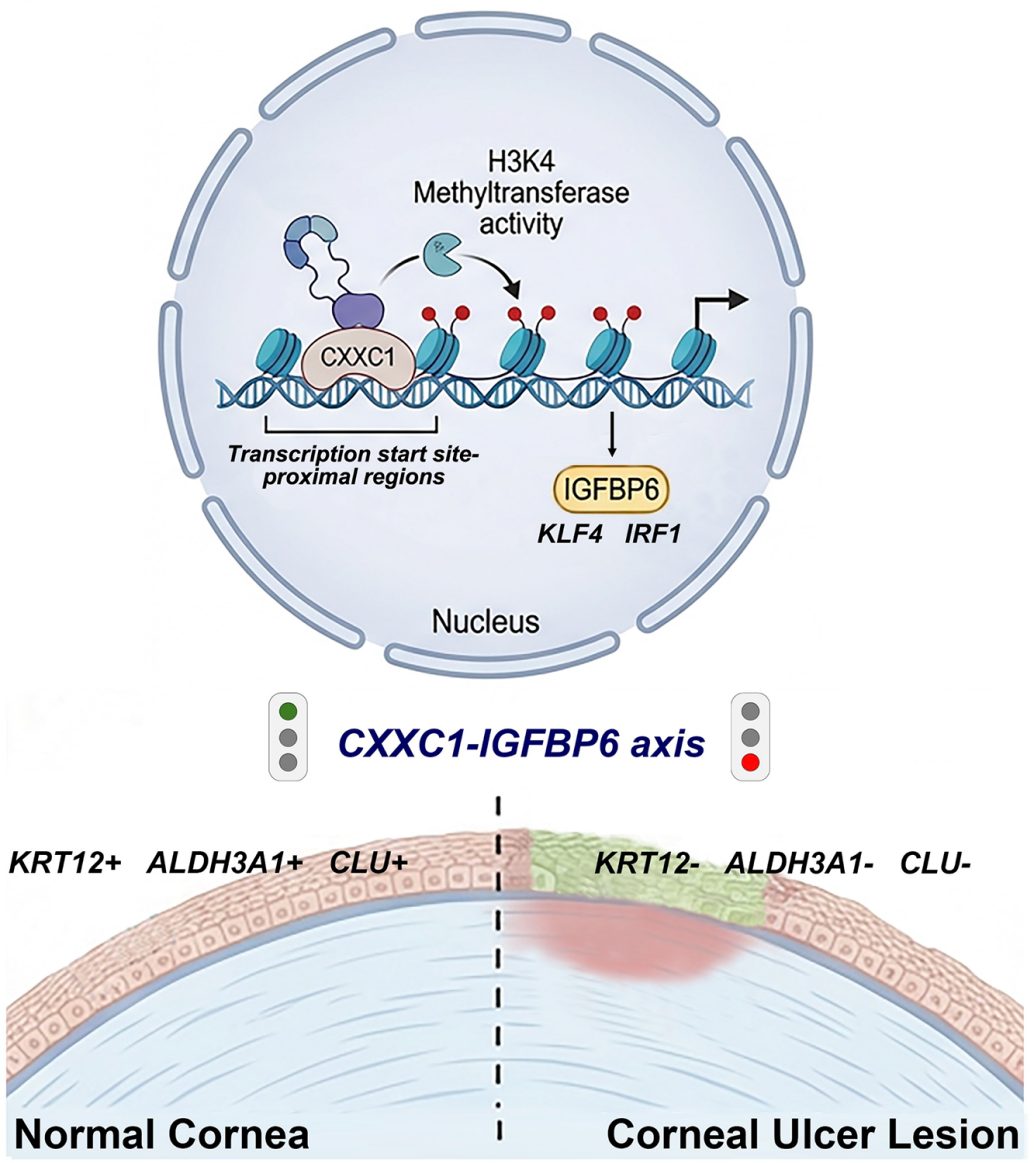
Schematic overview of CXXC1-IGFBP6 axis in corneal epithelium. CXXC1 promotes the expression of IGFBP6 and corneal identity factors by maintaining the appropriate H3K4me3 modification at transcription start site-proximal regions. The CXXC1-IGFBP6 axis supports CECs differentiation. Disruption of the CXXC1-IGFBP6 axis induces pathological corneal epithelial changes and is associated with corneal ulcer pathology.

Corneal ulcer is characterized by severe disruption of corneal epithelial integrity and differentiation status; therefore we further included corneal ulcer samples to examine whether the expression patterns of CXXC1, IGFBP6, and corneal differentiation-associated molecules identified in our in vitro system were similarly altered under pathological conditions. This analysis was intended to evaluate the clinical relevance of the CXXC1-IGFBP6 axis in corneal epithelial dysfunction. Compared with normal cornea and the non-ulcerated regions of corneal ulcer samples, immunofluorescence staining showed that CXXC1, IGFBP6, and the CEC-specific markers KRT12, ALDH3A1, and CLU were largely absent in the lesion areas of corneal ulcer tissues (Fig. 6A). These findings provide pathological evidence linking disruption of the CXXC1-IGFBP6 axis to corneal ulceration and are summarized in the schematic model shown in Figure 7.

## Discussion

Epigenetic modifications are essential for corneal differentiation and tissue function. We identify CXXC1 as a key epigenetic regulator required for corneal epithelium differentiation. Mechanistically, IGFBP6 is a direct transcriptional target of CXXC1, and CXXC1 sustains H3K4me3 at the IGFBP6 locus and at other loci encoding CEC differentiation regulators, thereby promoting their expression. Functionally, CXXC1-IGFBP6 axis contributes to CEC differentiation process, and loss of either component is associated with pathological corneal epithelial changes and disease. Therefore our study reveals a previously unrecognized epigenetic regulation mechanism governing corneal epithelial differentiation process and offers CXXC1 and IGFBP6 as potential therapeutic targets for corneal disorders.

Corneal epithelial differentiation and maintenance of epithelial identity have traditionally been attributed to lineage-specific transcription factors (TF) and their downstream regulators. Previous studies have shown that EHF, KLF4, and KLF7 are key determinants of regulating corneal identity and epithelial function.^18^^–^^20^ EHF cooperates with Krüppel-like factors (KLF4 and KLF5) to activate corneal epithelial genes and promote epithelial fate.^18^ Emerging evidence has also highlighted the role of specific TF-mediated epigenetic regulation in corneal differentiation process. FOXC1 modulated corneal epithelial genes expression by regulating H3K4me2 modification,^21^ whereas RORA reshapes histone modification to silence LSCs-specific genes and activate CECs-specific genes, thereby governing the LSC-to-CEC differentiation.^22^ However, little is known about the contribution of upstream chromatin-associated epigenetic regulators to corneal epithelial identity. Here, we identify CXXC1 as a key epigenetic regulator in corneal epithelium that promotes CECs differentiation by modulating IGFBP6 transcription and is associated with pathological corneal epithelial changes. By sustaining appropriate H3K4me3 levels at IGFBP6 and other corneal epithelial genes, CXXC1 fine-tunes their expression and further supports a non-keratinized epithelial state. Thus our work extends the current TF-based paradigm and provides new insight into how CXXC1-mediated epigenetic machinery required for corneal epithelial identity.

Insulin-like growth factor-binding protein 6 (IGFBP6) is a member of IGFBPs family which binds to insulin-like growth factors (IGFs) and modulates their biological activities. The secreted protein IGFBP6 binds IGF-II with preferential affinity over IGF-I, and has been implicated in the regulation of cell proliferation, differentiation, migration, angiogenesis, fibrosis, and the inflammatory response.^23^^–^^25^ However, its molecular role in the corneal epithelium remains unclear. In our study, depletion of *CXXC1* downregulated the expression of multiple IGFBPs, including IGFBP1, IGFBP2, IGFBP5, IGFBP6 and IGFBP7, indicating that CXXC1 broadly influences the IGFBP family at transcriptional level. Although knockdown of *IGFBP5* also decreased the expression of CECs-specific markers, ChIP-seq and H3K4me3 profiling revealed that CXXC1 selectively binds to IGFBP6 locus and directly sustains its expression through H3K4me3-dependent mechanisms. Taken together, IGFBP6 acts as a direct downstream target of CXXC1 in maintaining corneal identity and differentiation. Importantly, as a secreted protein, IGFBP6 may be more amenable to therapeutic manipulation than nuclear epigenetic regulators, therefore holds greater potential for future novel strategies in treatment of corneal diseases.

Our data also place within a broader regulatory network that converges on established corneal epithelial regulators. We found that CXXC1 binds to and regulates IRF1, which we previously showed to be downstream of FOXC1 in maintaining corneal identity and differentiation.^21^ Notably, depletion of *CXXC1* showed no impact on the expression of FOXC1. Reciprocally, silencing *FOXC1* did not alter CXXC1 levels as shown in RNA-seq data. These observations suggest that CXXC1 does not operate simply upstream or downstream of FOXC1, but instead may act in a partially parallel epigenetic pathway that converges on shared target genes such as IRF1. CXXC1 mainly shapes the permissive chromatin landscape through H3K4me3 at common loci. Dissecting how CXXC1 cooperates with FOXC1 and other corneal transcription factors will be an important goal for future studies and may further clarify how transcriptional and epigenetic cues are integrated to determine corneal epithelial fate.

Loss of *CXXC1* or *IGFBP6* in CECs induces a stress-associated transcriptional state characterized by activation of inflammatory, angiogenic, and hypoxia-responsive gene sets, which are commonly associated with corneal epithelial instability and pathological remodeling of the ocular surface.^26^^–^^29^ Consistent with this, CXXC1 and IGFBP6 are markedly diminished in human corneal ulcer lesions, suggesting that disruption of this epigenetic axis may contribute to impaired epithelial repair or, at minimum, serve as a molecular signature of disease-associated corneal instability.

Nonetheless, our study has several limitations. Most of our conclusions are derived from in vitro LSC-to-CEC differentiation models and *shRNA*-mediated knockdown, and in vivo validation using conditional *Cxxc1* or *Igfbp6* deletion in the corneal epithelium will be important to confirm the physiological relevance and temporal dynamics of this pathway. Moreover, although we identify IGFBP6 as a direct downstream effector of CXXC1, the precise IGFBP6-dependent signaling mechanisms and their contributions to inflammation, angiogenesis remains to be fully elucidated. Future work dissecting these downstream pathways, and testing whether restoration of IGFBP6 or pharmacologic modulation of the CXXC1-IGFBP6 axis can rescue corneal epithelial defects in vivo, will be critical to translate our mechanistic insights into therapeutic strategies for corneal disease.

## Supplementary Material

Supplement 1

Supplement 2
