# Supplementary material for: The CXXC1-IGFBP6 Axis Maintains Corneal Epithelial Differentiation via H3K4me3-Dependent Transcriptional Activation
Source: Invest Ophthalmol Vis Sci. 2026 Jul 2;67(8):7. doi: 10.1167/iovs.67.8.7 (PMC13332519; doi:10.1167/iovs.67.8.7)
Supplement: Supplement 1 [file iovs-67-8-7_s001.docx]

**Supplementary Figure 1:**

**
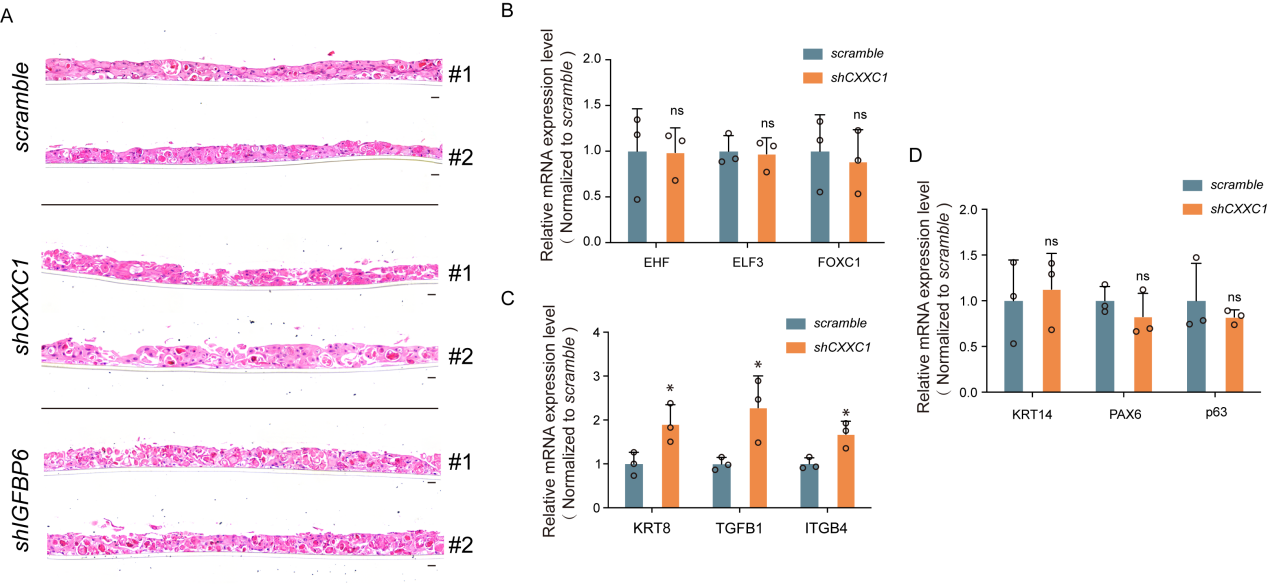
**

**(A)** H&E staining of differentiated CECs derived from *scramble*-, *shCXXC1*-, and *shIGFBP6*-transfected LSCs. *n* = 3. **(B)** qRT-PCR analysis of unchanged genes (*EHF*, *ELF3* and *FOXC1*) in differentiated CECs derived from *scramble*- or *shCXXC1*-transfected LSCs. *n* = 3. **(C)** qRT-PCR analysis of upregulated genes (*KRT8*, *TGFB1*, and *ITGB4*) in differentiated CECs derived from *scramble*- or *shCXXC1*-transfected LSCs. *n* = 3. **(D)** qRT-PCR analysis of LSC marker genes (*KRT14*, *PAX6*, and *p63*) in *scramble*- or *shCXXC1*-transfected LSCs. *n* = 3. **P* < 0.05.
