# Supplementary material for: The CXXC1-IGFBP6 Axis Maintains Corneal Epithelial Differentiation via H3K4me3-Dependent Transcriptional Activation
Source: Invest Ophthalmol Vis Sci. 2026 Jul 2;67(8):7. doi: 10.1167/iovs.67.8.7 (PMC13332519; doi:10.1167/iovs.67.8.7)
Supplement: Supplement 2 [file iovs-67-8-7_s002.docx]

**Supplemental tables**

**Supplementary Table S1. List of reagents**

| **Reagents for cell culture** | **Company or provider** | **Cat. no.** |
| --- | --- | --- |
| collagenase IV | Gibco | 17104019 |
| Trypsin-EDTA | Gibco | 25200072 |
| Matrigel | BD biosciences | 354230 |
| collagen I | Gibco | C3867 |
| PBS | ThermoFisher | C10010500BT |
| DMEM | Gibco | C11965500BT |
| DMEM/F12 | Gibco | C11330500BT |
| penicillin/streptomycin | Gibco | 10378-016 |
| knockout serum replacement | Gibco | 10828-028 |
| Cholera toxin | Sigma-Aldrich | C8052 |
| KGF | MCE | HY-P70597 |
| Calcium chloride | Sigma | 21115 |
| Retinoic acid | R&D | 0695 |
| polybrene | Millipore | TR-1003 |
| puromycin | Gibco | A11138-03 |
| **Reagents** | **Company** | **Cat. No.** |
| Hoechst 33342 | ThermoFisher | H3570 |
| histogel | ThermoFisher | #R904012 |
| iTaq™ Universal SYBR® Green Supermix kit | Bio-Rad | 1725124 |
| PrimeScript^TM^ RT Master Mix Kit | Takara | RR036B |
| RNeasy kit | Tiangen | DP451 |
| Protein A/G Dynabeads | Invitrogen | 10002D/10004D |
| MinElute PCR Purification Kit | QIAGEN | 28006 |
| VAHTS Universal DNA Library Prep Kit | Vazyme | ND607 |

**Supplementary Table S2. List of antibodies**

| **Antibody** | **Host Species** | **Company** | **Cat.No.** |
| --- | --- | --- | --- |
| KRT14 | Mouse | Invitrogen | MA5-11599 |
| PAX6 | Rabbit | Biolegend | 901301 |
| DeltaN p63 | Rabbit | Cell Signaling | 67825 S |
| KRT12 | Mouse | Santa Cruz biotechnology | sc515882 |
| ALDH3A1 | Rabbit | Gene Tex | GTX30042 |
| CLU | Rabbit | Proteintech | 12289-1-AP |
| CXXC1 | Rabbit | Abcam | ab198977 |
| IGFBP6 | Mouse | Proteintech | 67567-1-Ig |
| H3K4me3 | Rabbit | Cell Signaling | 9751S |
| GAPDH | Rabbit | Gene Tex | GTX100118 |

**Supplementary Table S3. List of primers**

| **Human Primers** |  | **Sequences (5’to 3’)** |
| --- | --- | --- |
| *GAPDH* | Sense | CTGGGCTACACTGAGCACC |
| *GAPDH* | Antisense | AAGTGGTCGTTGAGGGCAATG |
| *KRT12* | Sense | TTCCATGTTTGGTTCTAGTTCCG |
| *KRT12* | Antisense | TCATTGCCCGAGAGAATACCTA |
| *ALDH3A1* | Sense | TGTTCTCCAGCAACGACAAGG |
| *ALDH3A1* | Antisense | AGGGCAGAGAGTGCAAGGT |
| *CLU* | Sense | CCAATCAGGGAAGTAAGTACGTC |
| *CLU* | Antisense | CTTGCGCTCTTCGTTTGTTTT |
| *CXXC1* | Sense | GCAAACCGGACATCAACTGC |
| *CXXC1* | Antisense | GCACTCCCGACAGTACCAC |
| *IGFBP6* | Sense | TGTGAACCGCAGAGACCAAC |
| *IGFBP6* | Antisense | GCCCATCTCAGTGTCTTGGA |
| *KLF4* | Sense | CAGCTTCACCTATCCGATCCG |
| *KLF4* | Antisense | GACTCCCTGCCATAGAGGAGG |
| *IRF1* | Sense | ATGCCCATCACTCGGATGC |
| *IRF1* | Antisense | CCCTGCTTTGTATCGGCCTG |
| *EHF* | Sense | CAGTGCAGTAGTGACCTGTTC |
| *EHF* | Antisense | CTGTGCTACCATAGTTGGTGTC |
| *ELF3* | Sense | GGCCGATGACTTGGTACTGAC |
| *ELF3* | Antisense | GCTTGCGTCGTACTTGTTCTTC |
| *FOXC1* | Sense | TGTTCGAGTCACAGAGGATCG |
| *FOXC1* | Antisense | ACAGTCGTAGACGAAAGCTCC |
| *KRT8* | Sense | TCCTCAGGCAGCTATATGAAGAG |
| *KRT8* | Antisense | GGTTGGCAATATCCTCGTACTGT |
| *TGFB1* | Sense | CTAATGGTGGAAACCCACAACG |
| *TGFB1* | Antisense | TATCGCCAGGAATTGTTGCTG |
| *ITGB4* | Sense | CTCCACCGAGTCAGCCTTC |
| *ITGB4* | Antisense | CGGGTAGTCCTGTGTCCTGTA |
| *TP63* | Sense | GGACCAGCAGATTCAGAACGG |
| *TP63* | Antisense | AGGACACGTCGAAACTGTGC |
| *PAX6* | Sense | TGGGCAGGTATTACGAGACTG |
| *PAX6* | Antisense | ACTCCCGCTTATACTGGGCTA |
| *KRT14* | Sense | TGAGCCGCATTCTGAACGAG |
| *KRT14* | Antisense | GATGACTGCGATCCAGAGGA |

**Supplementary Table S4. List of shRNA sequences**

| **shRNA** | **Sequences (5' To 3')** |
| --- | --- |
| *scramble* | 5′-CCTAAGGTTAAGTCGCCCTCG-3′ |
| *shCXXC1-1* | 5′-GCACAAGGATAAATGGAAACA-3′ |
| *shCXXC1-2* | 5′-CATCCGGATCACTGAGAAGAT-3′ |
| *shIGFBP6-1* | 5′-GAGAATCCTAAGGAGAGTAAA-3′ |
| *shIGFBP6-2* | 5′-CGCAGAGACCAACAGAGGAAT-3′ |
